# A Dynamic Task Allocation Algorithm for Heterogeneous UUV Swarms

**DOI:** 10.3390/s22062122

**Published:** 2022-03-09

**Authors:** Xiaojun Wu, Zhiyuan Gao, Sheng Yuan, Qiao Hu, Zerui Dang

**Affiliations:** 1 School of Software Engineering, Xi’an Jiaotong University, Xi’an 710049, China; gzy1993619123@stu.xjtu.edu.cn (Z.G.); jiaqi_vector@stu.xjtu.edu.cn (Z.D.); 2 Shaanxi Key Laboratory of Intelligent Robots, Xi’an Jiaotong University, Xi’an 710049, China; hqxjtu@xjtu.edu.cn

**Keywords:** unmanned underwater vehicle, dynamic task allocation, auction algorithm, multiple intelligent agents, decentralized system

## Abstract

Aiming at the task allocation problem of heterogeneous unmanned underwater vehicle (UUV) swarms, this paper proposes a dynamic extended consensus-based bundle algorithm (DECBBA) based on consistency algorithm. Our algorithm considers the multi-UUV task allocation problem that each UUV can individually complete multiple tasks, constructs a “UUV-task” matching matrix and designs new marginal utility, reward and cost functions for the influence of time, path and UUV voyage. Furthermore, in view of the unfavorable factors that restrict the underwater acoustic communication range between UUVs in the real environment, our algorithm complete dynamic task allocation of UUV swarms with optimization in load balance indicator by the update of the UUV individual and the task completion status in the discrete time stage. The performance indicators (including global utility and task completion rate) of the dynamic task allocation algorithm in the scenario with communication constraints can be well close to the static algorithm in the ideal scenario without communication constraints. The simulation experiment results show that the algorithm proposed in this paper can quickly and efficiently obtain the dynamic and conflict-free task allocation assignment of UUV swarms with great performance.

## 1. Introduction

As a new type of underwater vehicle, the unmanned underwater vehicle (UUV) is one of the effective tools for seabed exploration, with advantages of intelligence and being unmanned. With the increasing complexity of underwater tasks, a single UUV is greatly restricted in terms of computing, endurance, and capability, and can no longer meet the increasingly complex and diversified demands of underwater tasks. In such a context, the formation of multiple UUVs into swarms for collaborative operations comes into being to make up for the deficiencies of single UUV through effective coordination.

**Figure 1:**
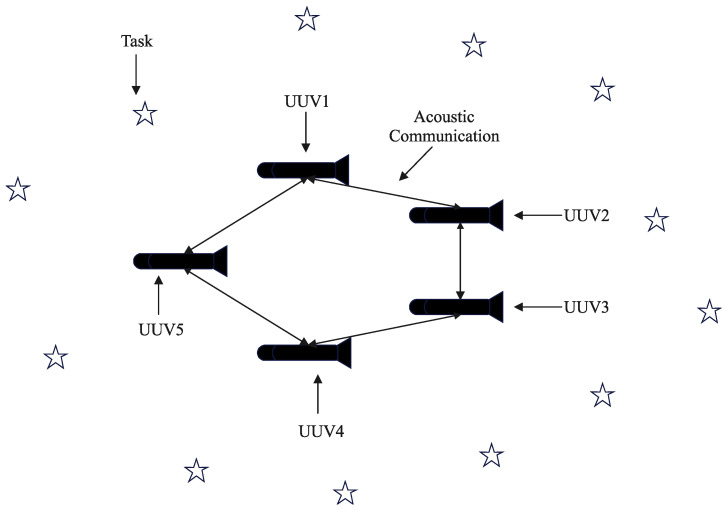
UUV swarm network.

Task allocation is a very important stage in the process of UUV swarms completing underwater tasks, and different assignment schemes are directly related to the performance of task completion. The purpose of collaborative task allocation for multiple UUV swarms is to find a better solution to plan the trajectories of individual UUVs to accomplish underwater detection, tracking, and rescue tasks under the constraints of range and time, so as to make the global utility greater, the cost smaller, and the cost effectiveness higher. Figure 1 illustrates a conceptual schematic of a UUV swarm network.

The task allocation of UUV swarms belongs to the category of multi-agent collaborative task allocation problems which are limited by objective factors such as environment, resources and communication conditions, and subjective factors such as mathematical models, objective functions, and constraint conditions. Classical models of multi-agent task allocation problems include the traveling salesman problem (TSP), the vehicle routing problem (VRP), the multi-dimensional multiple choice knapsack problem (MMKP), etc. The task allocation problem of UUV swarms studied in this paper is a kind of nonlinear combinatorial optimization problem which is difficult to solve by polynomial time complexity algorithms. The solutions to this type of problem can be divided into centralized and decentralized methods, including game theory methods, heuristic algorithms, reinforcement learning (RL), auction-based algorithms, etc.

In centralized methods, agents communicate their situation awareness (SA) to a central planner, which generates a plan for the entire swarm. Sun et al. established a three-level model including command level, mission planning level, and motion planning level for multiple UUVs visiting a set of underwater sites in a large-scale and time-varying environment, and implemented a real-time task planning by selecting heuristic algorithms based on the characteristics of the different levels [1]. This type of methods places more of the heavy processing requirements on the central planner, thus making the agents smaller and cheaper to build with lower cost. However, agents must consistently communicate with a fixed central planner, thus reducing the range of tasks that the swarm can handle, and easily creating a single point of failure.

While in decentralized methods, each agent, as an independent unit, can make autonomous decisions about processing and communication, thus providing greater fault tolerance, flexibility, and reliability [2]. The main problem in decentralized methods is how to deal with the coupling relationships between individual agents. Therefore, it is crucial to design appropriate consistency protocols to ensure constraint feasibility and maximum utility. The content and rules of information communicated between agents must be specified in consistency protocols. Kumar et al. presented a network model for drone assisted IoT environment, employed Neuro-fuzzy interference system to jointly combine relative velocity of drones, expected link availability period, residual route load capacity and route delay, and developed a drone assisted distributed routing framework, which can be utilized for any applications of drone assisted ad hoc networking [3]. Furthermore, they built an UAV-centric mobility model to develop a quality of service provisioning framework for a UAV-assisted aerial ad hoc network environment (QSPU) and proved that QSPU outperforms the state-of-the-art protocols [4].

Game-theoretic tools constitute one of the most natural methods to task allocation problems for multiple agents. Their basic idea is that agents act as separate decision-making entities to maximize their local utilities based on their knowledge of the environment and other agents. They are applicable to modeling in non-cooperative environments and are particularly suitable for computing mutually agreeable task allocations in potential games where the task allocation profile is shown to converge to a Nash equilibrium (NE) [5]. Jang et al. proposed a novel game-theoretical autonomous decision-making framework to address a task allocation problem for a swarm of multiple agents and guaranteed at least 50% of sub-optimality [6]. Bakolas et al. proposed a decentralized game theoretic framework for multi-agent dynamic task allocation problems, which the agents negotiate with each other to find a mutually agreeable task allocation profile based on evaluations of the task utilities that reflect their current states [7]. Jin et al. formulated the task allocation problem as a potential game with the goal of maximizing task utility, then confirmed the existence of Nash Equilibrium and proposed a concurrent best response algorithm with rapid convergence to achieve the desirable solution [8]. Although game theory is an important tool for extending the task allocation problem to multi-agent, convergence to an effective Nash equilibrium is not always guaranteed in all scenarios and can be computationally expensive.

Heuristics algorithms include evolutionary algorithms, swarm intelligence algorithms and other intelligent optimization algorithms, which use modern intelligent optimization algorithms to optimize task planning to achieve a balance between minimum solving time and optimal solution. Yavuz et al. combined the clustering algorithm, Hungarian algorithm, and ant colony algorithm, and proposed an approach to efficiently perform the task assignment problem in cases where the number of targets and UAVs is large [9]. Wu et al. used the self-adjusting characteristics of the bee colony to achieve task allocation [10]. However, the heuristic algorithm also has several disadvantages, such as the inability to avoid falling into local optima, lacking effective iterative stopping conditions, and the great influence of parameter settings on the algorithm.

Machine learning algorithms are also increasingly used in task allocation problems because they can process large amounts of information through neural networks and handle unknown environments via reinforcement learning (especially deep Q-learning) [11]. Bakshi et al. applied Recurrent Neural Network (RNN) in scheduling problems to schedule autonomous mobile robots (AMRs) in a timely manner such that a large school of AMRs can finish all the assigned tasks within the shortest time [12]. Aiming at the multi-task allocation problem in research of autonomous underwater vehicles (AUV), Zhu et al. proposed a multi-AUV multi-target assignment strategy based on Self-Organization Mapping (SOM) neural network [13]. However, these methods are more computationally intensive and require high computing resource capacity.

Auction algorithm based on Contract Network Protocol (CNP) is a distributed intelligent optimization algorithm, including four steps: announcing–bidding–winning–signing. Each agent member in the processing lifecycle can make independent decisions and connect through communications. Otte et al. studied the multi-robot task allocation in large-scale ocean environment with limited communication and compared the performance of six auction algorithms by simulation experiments [14]. Based on contract network mechanism, Choi et al. proposed a consensus-based auction algorithm (CBAA) to solve the single task allocation problem. Meanwhile, a consensus-based bundle algorithm (CBBA) was developed further to solve the multi-task allocation problem [15]. Both algorithms can achieve better conflict-free solutions. Since CBBA was proposed, many scholars have tried to improve it for solving more complex problems. Aiming at the problem of on-orbit assembly task assignment for large-scale spatial structure, Yu et al. proposed an auction algorithm based on extended CBBA to ensure the successful allocation of complex on-orbit assembly tasks [16]. Bertuccelli et al. improved CBBA by using Dijkstra algorithm, which could solve the problems of path collision avoidance and task allocation [17]. By dividing UAV and tasks into several clusters, Cui and Majeed et al. reduced the overall computational cost [18,19]. Braquet et al. proposed a decentralized auction-based algorithm for the solution of dynamic task allocation problems for spatially distributed multi-agent system, however, they considered the problem domain of multiple agents working together to complete a single task, i.e., there was no task conflict between agents [20]. Considering the emergence of new tasks in battlefields, Buckman et al. proposed a partial replanning algorithm (CBBA-PR). This algorithm takes out partially allocated tasks from the allocation state, and then mixes them with new tasks for reallocation purposes, thereby achieving the goal of ensuring optimality and real-time performance in dynamic allocation, however, they did not take into account the heterogeneity of agents and the different types of tasks [21]. Zhang et al. introduced a new dynamic task generation mechanism that satisfied the task timing constraints, developed a new method for reconstructing partial paths to achieve optimal allocation profile, and imported the asynchronous task allocation mechanism to reduce the time and communication cost of the algorithm [22].

As will be readily seen, current research on the task allocation of multi-agent systems has achieved a lot of results, especially the many improvements to CBBA. However, the existing related research and methods have the following shortcomings: (1) the current research mainly focuses on unmanned aerial vehicles (UAV) or robot swarms, and there are few related studies on UUV swarms. In particular, given the special characteristics of the underwater environment, solving the task allocation problem of UUV swarms is greatly restricted by the underwater acoustic communication conditions. (2) Most of the current studies focus on the static task allocation problem, i.e., the task is assigned only once before agents starting to complete the task, and the optimal solution cannot be obtained through subsequent adjustments. Therefore, this paper proposes a dynamic task allocation algorithm based on extended CBBA. The contributions in this paper are as follows:

- This paper takes the task allocation of heterogeneous UUV swarms as the main scenario, considers the influence of factors such as time, path and UUV voyage on marginal utility, and designs new marginal reward, cost and utility functions to further optimize CBBA.
- In view of the unfavorable factors that restrict the underwater acoustic communication range between UUVs in the real environment, this paper completes dynamic task allocation of UUV swarms by the update of the UUV individual and task completion status in the discrete time stage with optimization in load balance indicator.

Outline: The rest of the paper is presented as follows. We discuss the problem description and mathematical model in Section 2. We address the static task allocation problem identify the cost, reward and utility function in Section 3. The proposed dynamic task allocation algorithm is presented in Section 4. In Section 5, we present simulation experiments. Finally, concluding remarks and directions for future work are provided in Section 6.

## 2. Problem Description and Mathematical Model

Considering a UUV swarm $U:=\left\{U_{1},U_{2},\cdots ,U_{N_{U}}\right\}$ composed of $N_{U}$ heterogeneous UUVs and a task set $T:=\left\{T_{1},T_{2},\cdots ,T_{N_{T}}\right\}$ of $N_{T}$ different type tasks, different types of UUVs can complete one or more different types of tasks, i.e., the matching matrix between UUVs and tasks has the following element.

(1) $${\mathrm{Match}}_{ij}=\{\begin{array}{c} 1,U_{i}\;can\;complete\;T_{j}\\ 0,\;otherwise \end{array}$$

Finds a conflict-free task allocation profile that assigns each task to at most one UUV, completes as many tasks as possible, and minimizes the required path cost to maximize the global utility. Correspondingly, the task allocation problem model of the entire UUV swarm can be defined as follows.

(2) $$f=\max\overset{N_{U}}{\underset{i=1}{\sum }}\overset{N_{T}}{\underset{j=1}{\sum }}r_{ij}\left(A_{i},P_{i}\right)$$

(3) $$\overset{N_{T}}{\underset{j=1}{\sum }}a_{ij}\leq B_{i}$$

(4) $$\overset{N_{U}}{\underset{i=1}{\sum }}a_{ij}\leq 1$$

(5) $$\overset{N_{U}}{\underset{i=1}{\sum }}\overset{N_{T}}{\underset{j=1}{\sum }}a_{ij}\leq \min\left(N_{T},\overset{N_{T}}{\underset{i=1}{\sum }}B_{i}\right)$$

(6) $$D\left(P_{i}\right)\leq D_{i},\mathrm{for}\;i=1,2,\cdots ,N_{U}$$

Formula (2) represents the global objective function f. $A_{i}\in {\left\{0,1\right\}}^{T}$ is the binary decision vector of $U_{i}$. When the task $T_{j}$ is assigned to $U_{i}$, the jth element of $A_{i}$ is $a_{ij}=1$, otherwise is $a_{ij}=0$. $P_{i}\in {\left(\left\{1,2,\cdots ,N_{T}\right\}\cup \emptyset \right)}^{B_{i}}$ denotes the task completion path sequence of $U_{i}$, and $B_{i}$ represents the maximum number of tasks that $U_{i}$ can complete. When the kth element of $P_{i}$ is $j\in \left\{1,2,\cdots ,N_{T}\right\}$, it indicates that $U_{i}$ performs $T_{j}$ at the kth position in its path, and when it is ∅, it indicates that $U_{i}$ has no task to perform at the kth and subsequent positions, $r_{ij}\left(A_{i},P_{i}\right)\geq 0$ denotes $T_{j}$’s utility obtained when it is performed by $U_{i}$ at the kth position in its path, usually related to path factors, such as the task completion path cost and the task completion time. The purpose of the entire task allocation problem of UUV swarms is to determine the task allocation decision vector $A_{i}$ and path vector $P_{i}$ of each UUV so as to maximize the global utility.

Formulas (3)–(6) express constraint conditions. Formula (3) represents the maximum number of tasks that $U_{i}$ can complete. Formula (4) shows that each task can only be performed by at most one UUV. Formula (5) limits the total number of tasks that can be completed by all UUVs. In Formula (6), $D\left(P_{i}\right)$ represents the path cost that $U_{i}$ needs to pay to perform the task sequence along the path $P_{i}$, and $D_{i}$ represents the maximum voyage of $U_{i}$.

**Figure 2:**
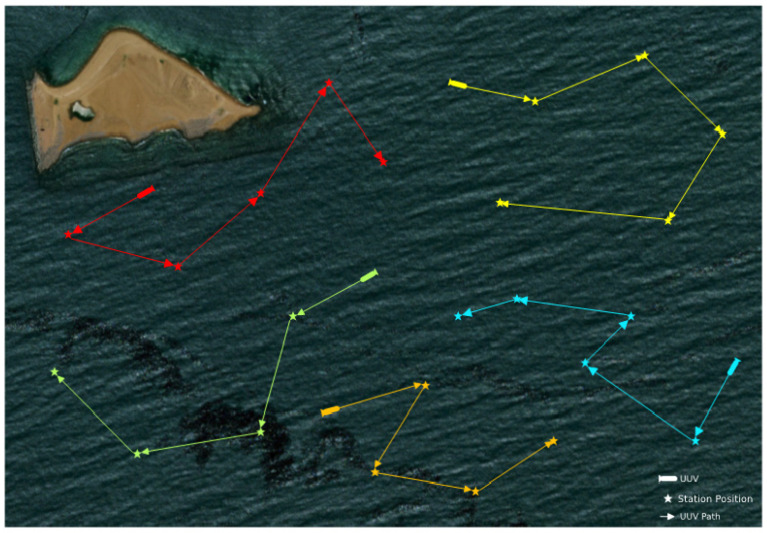
Diagram of a UUV swarm visiting multiple underwater stations.

As shown in Figure 2, the above model can be used to solve the allocation problem of a UUV swarm visiting multiple underwater stations in a large area of sea.

## 3. Static Task Allocation Algorithm

Aiming at the static task allocation problem of complex multi-agent swarms, the CBBA proposed by Choi et al. can converge to a conflict-free task allocation profile with 50% optimality. The efficiency of the algorithm is related to the number of agents and tasks, and it is robust to inconsistent situational awareness of the entire swarm and changes in the communication network topology. However, the original CBBA does not sufficiently consider the impact of time and path on task utility and marginal utility in the actual task allocation problem of UUV swarms. Therefore, this paper first optimizes the utility function (reward and cost functions).

Similar to the original CBBA, the extended consensus-based bundle algorithm (ECBBA) proposed in this paper consists of two phases of iteration: the task bundle construction phase and the conflict resolution phase.

The first phase is the task bundle construction, where ECBBA creates a sequence of task bundles for each UUV. As the allocation process is continuously updated, each UUV keeps adding tasks to its task bundle until there are no more tasks to add or the upper limit *B* for the number of tasks it can complete is reached. Note that task allocation is performed only once in the static allocation problem, so all UUVs have the same upper bound on the number of tasks and remain constant during the allocation process.

The task bundle of $U_{i}$ includes two vectors, namely the task sequence $b_{i}$ and the path $p_{i}$. Tasks in $b_{i}$ are sorted by the order in which they were added, and $p_{i}$ indicates the order in which $U_{i}$ will perform the tasks in $b_{i}$. The length of $b_{i}$ and $p_{i}$ cannot exceed the upper limit B for the number of tasks $U_{i}$ can complete. $S_{i}^{p_{i}}$ represents the utility obtained by $U_{i}$ performing $T_{j}$ along $p_{i}$. $p_{i}{⊕}_{n}\left\{j\right\}$ represents that task $T_{j}$ will be inserted into the nth position in $p_{i}$, and $S_{i}^{p_{i}{⊕}_{n}\left\{j\right\}}$ represents the new utility after inserting $T_{j}$ into the nth position in $p_{i}$. When $T_{j}$ is added to $b_{i}$, the utility increment, i.e., the marginal utility value $\Delta S_{ij}$ is

(7) $$\Delta S_{ij}=\{\begin{array}{c} 0,\;j\in b_{i}\\ \max_{n\leq \left|p_{i}\right|}S_{i}^{p_{i}{⊕}_{n}\left\{j\right\}}-S_{i}^{p_{i}},otherwise \end{array}$$

In other words, ECBBA inserts the new task into the location in the path with the highest utility increment, and that utility increment becomes the marginal utility value associated with that task for a given current path. Accordingly, if the task is already included in the path, its marginal utility will be zero.

The marginal utility value of $T_{j}$ can be expressed as a function of the marginal reward value and the marginal cost.

The range constraint is not considered in the original CBBA. However, in the actual task allocation problem of UUV swarms, the UUV’s voyage is an important factor affecting its capability to complete the assigned task. Therefore, in this paper, the limit of $U_{i}$’s voyage is set to *D_i_* in Formula (8), which means that when adding tasks to the task sequence and paths in the bundle construction phase, it is necessary to ensure that the path cost after $T_{j}$ is inserted into the nth position of path $p_{i}$ does not exceed the voyage constraint $D_{i}$, i.e.,

(8) $$D\left(p_{i}{⊕}_{n}\left\{T_{j}\right\}\right)<D_{i}$$

In addition, the original CBBA only considers the task’s time-discounting reward when designing the marginal reward function, i.e., $R_{j}^{p_{i}}={\bar{r}}_{j}\cdot e^{-\lambda_{j}\cdot \tau_{i}^{j}\left(p_{i}\right)}$, where $\lambda_{j}<1$ is time-discounting factor for task $T_{j}$, ${\bar{r}}_{j}$ is the static reward value for task $T_{j}$, and $\tau_{i}^{j}\left(p_{i}\right)$ is the time it takes for $U_{i}$ to travel along path $p_{i}$ to reach the location of task $T_{j}$. However, when task $T_{j}$ is inserted into the path, the marginal reward value of tasks in subsequent positions in the path is affected accordingly, which is not considered in the original CBBA. Therefore, in order to improve the accuracy of the marginal reward value, the ECBBA in this paper designs the marginal reward function $R_{j}^{p_{i}}$ as

(9) $$R_{j}^{p_{i}}={\bar{r}}_{j}\cdot e^{-\lambda_{j}\cdot \tau_{i}^{j}\left(p_{i}\right)}+\sum \Delta R_{m}^{p_{i}}$$

In Formula (9), $\Delta R_{T_{m}}^{p_{i}}$ is the marginal increment of $T_{m}$ after inserting task $T_{j}$ into the path, and $T_{m}$ is the task added to the task sequence before $T_{j}$, but located after $T_{j}$ in the path. Due to the decreasing nature of the marginal function, the marginal rewards of other tasks in subsequent positions tend to decrease.

Furthermore, when considering the marginal cost, the original CBBA only calculates the distance cost between position of $T_{j}$ and the initial position of $U_{i}$, i.e., $cost_{T_{j}}^{p_{i}}=Fi\cdot D(U_{i},T_{j})$, where $F_{i}$ is the fuel consumption per unit mile of $U_{i}$. However, the marginal cost is essentially related to the state of $U_{i}$ (actual position in the path). In order to improve the accuracy of the marginal cost function, it is necessary to calculate the marginal cost as the additional path cost required to complete $T_{j}$. Therefore, when inserting $T_{j}$ into the nth position of $p_{i}$, the marginal cost function $cost_{T_{j}}^{p_{i}}$ designed by ECBBA in this paper is

(10) $$cost_{T_{j}}^{p_{i}}=F_{i}\cdot \left(D(p_{i}{⊕}_{n}\left\{T_{j}\right\}\right)-D(p_{i}))$$

Considering factors such as reward and cost normalization and UUV voyage, the marginal utility function $\Delta S_{ij}$ of ECBBA in this paper is expressed as follows.

(11) $$\Delta S_{ij}=R_{j}^{p_{i}}\left(1-cost_{T_{j}}^{p_{i}}/\left(F_{i}\cdot D_{i}\right)\right)$$

Task bundles constructed by the UUV swarm in the first stage are based on local information, so there are inevitably many conflict situations between task sequences of different UUVs, and conflict-free allocation profile needs to be achieved by appropriate conflict resolution rules. At this point, UUVs need to know not only their marginal utilities for the associated tasks, but also the allocation information of other UUVs. Once task bundles of all UUVs are constructed, it is necessary to construct the bidding vector for the second stage of conflict resolution, including the biding UUV vector $z_{i}$ and the biding utility vector $y_{i}$. $z_{i}$ denotes the UUV with the highest marginal utility for completing tasks in $U_{i}$’s cognition, and $y_{i}$ denotes the marginal utility value corresponding to $z_{i}$.

The second phase is the conflict resolution phase. In this phase, besides the two vectors $y_{i}$ and $z_{i}$ constructed above, it is also necessary to construct a communication timestamp vector $s_{i}$, which represents the time information of $U_{i}$ communicating with other UUVs. In each communication step, the timestamp vector s is updated as follows.

(12) $$s_{ik}=\{\begin{array}{c} \tau_{r},g_{ik}=1\\ \max_{m:g_{im}=1}s_{mk},otherwise \end{array}$$

In Formula (12), $\tau_{r}$ is the message reception time and $g_{ik}=1$ indicates that $U_{i}$ and $U_{k}$ are neighbor nodes in the communication network G. When $U_{i}$ receives a message from $U_{k}$, vectors $y_{i}$, $z_{i}$ and $s_{i}$ are used to resolve the conflicts between the task sequences of UUVs. For task $T_{j}$, $U_{i}$ may take three actions: (1) update: $y_{ij}=y_{kj},z_{ij}=z_{kj}$; (2) reset: $y_{ij}=0,z_{ij}=0$; and (3) leave: $y_{ij}=y_{ij},z_{ij}=z_{ij}$. The specific conflict resolution rules are shown in Table 1.

After resolving the conflicts in task sequences according to the rules in the above table, all UUVs check whether there are tasks that need to be updated or reset in their task bundles, and if so, release them and all tasks added to the task bundle after them. After doing that, the ECBBA returns to the first phase. Accordingly, there is iterating between these two phases until it converges into a conflict-free task allocation profile.

## 4. Dynamic Task Allocation Algorithm

In real underwater environments, the main way of communication between UUVs is hydro-acoustic communication. Due to the multipath effect and time-varying effect of the hydro-acoustic communication channel, its available frequency band is narrow and the signal attenuation is serious, especially in long distance transmission. Compared with the UAV swarm whose converge distance can reach tens or even hundreds of kilometers, the communication distance between UUVs often cannot exceed 1 km under the premise of ensuring an effective communication bandwidth.

The original CBBA needs to ensure that there is no communication range constraint among agents to assign tasks. When considering the fact that communication range is constrained, agents in the CBBA or the ECBBA can only communicate with neighbor agents whose initial position is within their own communication range to resolve task conflicts after constructing task bundles, which inevitably results in that some task assignment conflicts cannot be resolved, and one task may be assigned to different agents. In addition, the task allocation profile cannot be dynamically adjusted, thus leading to a serious waste of resources and affecting the global utility and performance.

Based on the above critiques, this paper transforms the static task allocation problem into a dynamic one, and proposes a dynamic extended consensus-based decentralized bundle algorithm (DECBBA). The main idea of DECBBA is to transform the entire allocation process into multiple static task allocations under discrete time steps, i.e., the motion process of the UUV swarm is discretized into time steps T. When T=0, UUVs converge to a task allocation profile with partial conflict by iterating between the bundle construction phase and the conflict resolution phase under the constraint of communication range $D_{com\;dis}$ based on initial position information of themselves and tasks. Successively, all UUV individuals follow the planned task sequence and path with the increase of discrete time step toward the current task target position. The communication network topology state of the UUV swarm changes when different UUVs enter the communication range, or the task state changes when a task is completed. The UUVs exchange path and bidding information with other UUVs within the communication range according to the current state of themselves and tasks, resolve the task conflicts in previous allocation profile, reassign tasks, and update the time step T←T+1. Repeat the above process until all UUVs have completed all tasks in their task sequence or exceed the maximum task completion time $T_{\max}$. The specific process is shown in Algorithm 1.
**Algorithm 1:** Dynamic Extended CBBA (DECBBA).1:$\;\mathrm{INPUT}:\;T=0,G\left(0\right)=O,POS_{i}\left(0\right),POS_{j},P_{i}\left(0\right)=\emptyset$$,\;T_{\max}$2:$\;\mathrm{WHILE}\;T<T_{\max}$ AND T=0$\;\mathrm{OR}\;P_{i}\left(T\right)\neq \emptyset$ **DO**3:  FOR i=1$\;\mathrm{to}\;N_{U}$4:   FOR j=1$\;\mathrm{to}\;N_{U}$5:    IF i≠j$\;\mathrm{AND}\;D\left(U_{i},U_{j}\right)\leq D_{com\;dis}$6:$\;\;\;\;\;\;g_{ij}\left(T\right)=1$7:$\;\;\;\;\;\;g_{ji}\left(T\right)=1$8:      **ELSE**
9:$\;\;\;\;\;\;g_{ij}\left(T\right)=0$10:$\;\;\;\;g_{ji}\left(T\right)=0$11:      **END**
12:    **END**
13:  **END**
14:    IF G(T)≠G(T−1)15:   FOR i=1$\;\mathrm{to}\;N_{U}$16:$\;\;\;\;U_{i}$$\;\mathrm{build}\;\mathrm{task}\;\mathrm{bundles}\;b_{i}$$\;\mathrm{and}\;p_{i}$ with highest marginal utility
17:$\;\;\;\;U_{i}$$\;\mathrm{resolute}\;\mathrm{task}\;\mathrm{conflicts}\;\mathrm{with}\;U_{k}$ in its communication range
18:    **END**
19:  **END**
20:    FOR i=1$\;\mathrm{to}\;N_{U}$21:$\;\;\;T_{j}=P_{i}^{1}\left(T\right)$22:$\;\;\;\mathrm{IF}\;D\left(U_{i},T_{j}\right)\leq \left|V_{i}\left(T\right)\right|\cdot \Delta T$23:$\;\;\;\;P_{i}\left(T+1\right)=P_{i}^{2:\left|P_{i}\right|}\left(T\right)$24:$\;\;\;\;POS_{i}\left(T+1\right)=POS_{j}$25:    **ELSE**
26:$\;\;\;\;POS_{i}\left(T+1\right)=POS_{i}\left(T\right)+V_{i}\left(T\right)\cdot \Delta T$27:    **END**
28:  **END**
29:    T=T+130:**END**

**Figure 3:**
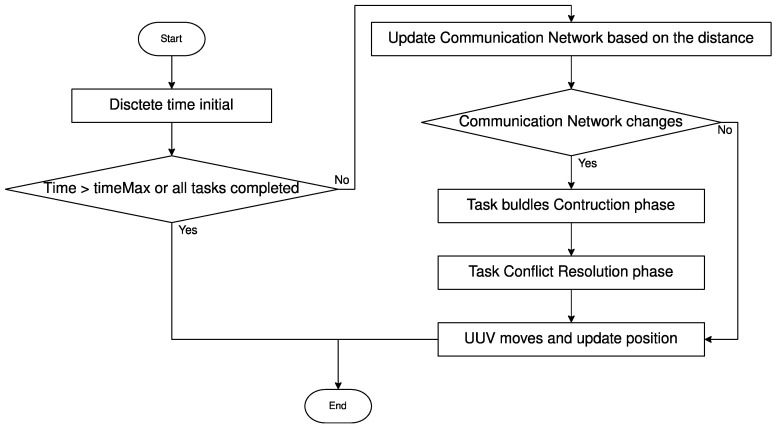
Flowchart of algorithm.

In Algorithm 1, T is the discrete time step, G is the communication network of UUV swarms, $POS_{i}\left(t\right)$ denotes the real-time location of $U_{i}$, $POS_{j}$ denotes the location of task $T_{j}$, and $P_{i}\left(t\right)$ denotes the real-time path sequence of $U_{i}$. Line 1 represents the initial moment input value of algorithm, lines 2–30 show the whole dynamic task allocation process. Lines 3–13 indicate that at each discrete time step, the UUV swarm updates its communication network based on the distance. Lines 14–19 illustrate that when the communication network changes, the UUV swarm reiterates the bundle construction and task conflict resolution process. Lines 15–20 indicate that at each time step, each UUV moves along its path and updates its position. The flowchart of the algorithm is shown in Figure 3.

In both the original CBBA and the ECBBA proposed in this paper, the upper limit B of the number of tasks for UUVs is a fixed value. An unreasonable value of B will lead to a situation where some UUV’s task sequence paths are too long or too short, i.e., unbalanced swarm load, and will make the total task completion time longer. In contrast, in the DECBBA, UUVs can dynamically adjust their own task number upper limit according to the task sequence length of neighboring UUVs in the communication network, thus improving the load balancing rate and reducing task completion time.

Convergence and Complexity: In the CBBA, $N_{U}$ UUVs can agree on the allocation profile by iterating $N_{U}D_{\max}$ times on a static communication network of maximum diameter $D_{\max}$ when the utility function satisfying diminishing marginal gain condition (DMG), i.e., the value of a task does not increase as other elements are added to the set before it. In the ECBBA, the utility function still satisfies the DMG condition. Therefore, it can still converge to a conflict-free solution in a finite time $N_{U}D_{\max}$. In the DECBBA, although the communication network G varies with the discrete time step T, convergence can be guaranteed in finite time at each iteration time step, and then the whole dynamic algorithm can converge in at most a finite time $N_{U}D_{\max}T_{\max}$. In the ECBBA proposed in this paper, it is necessary to store the information related to each UUV individual and task, the communication state among UUVs, the UUV task sequence $b_{i}$, the UUV path sequence $p_{i}$, the biding UUV vector $z_{i}$ and the biding utility vector $y_{i}$. Depending on the actual needs of underwater tasks, the number of tasks $N_{T}$ is often more than the number of UUVs $N_{U}$, so the space complexity of the ECBBA is $O\left(N_{U}\cdot N_{T}\right)$. Similarly, in the DECBBA, the above information needs to be stored at each discrete time step, so the space complexity of DECBBA is $O\left(N_{U}\cdot N_{T}\cdot T_{\max}\right)$.

## 5. Simulation and Results

In this section, the effectiveness of both the ECBBA and the DECBBA is demonstrated through simulation experiments, respectively. The simulation experiments assume that multiple heterogeneous UUVs are required to perform several different types of tasks in a two-dimensional area of 10 km × 10 km. The maximum simulation discrete time step $T_{\max}$ is 10,000 s.

Three heterogeneous UUVs (portable, light and heavy) with three different task types (detect, track and rescue) are set up in this paper. Each UUV has a different velocity $V_{i}$, voyage $D_{i}$, and fuel consumption per unit mile $F_{i}$. Each task also has its own duration $t_{j}$, static reward ${\bar{r}}_{j}$, and time-discounting factor $\lambda_{j}$.

Parameters of UUVs and tasks are set as shown in Table 2 and Table 3, respectively.

The UUV and task matching types are shown in Table 4.

Based on the above parameters, simulation experiments are performed by using MATLAB R2021b software on a PC with Intel(R) Core(TM) i5-9500 3.00 GHz CPU, 16.0 GB RAM, and Windows 10 professional operating system.

**Figure 4:**
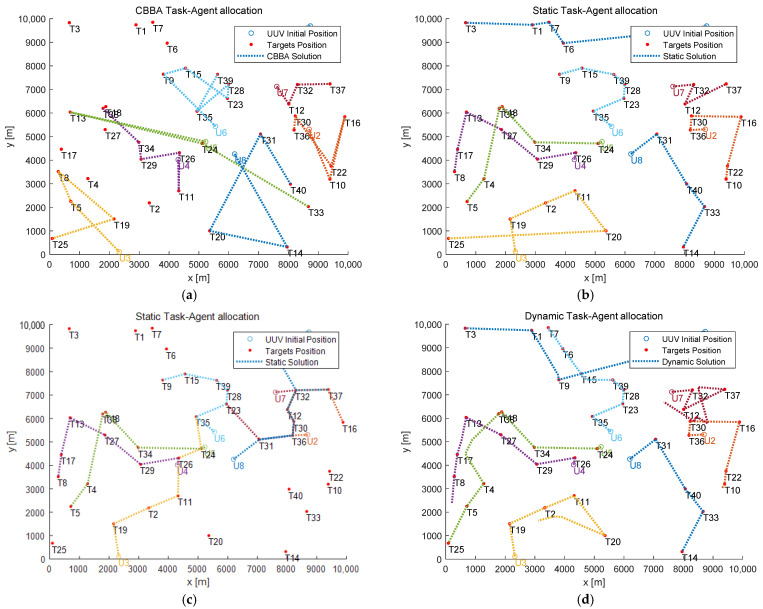
Task allocation profile. (**a**) Original CBBA (without communication range constraint). (**b**) ECBBA (without communication range constraint). (**c**) ECBBA (with communication range constraint). (**d**) DECBBA (with communication range constraint).

**Figure 5:**
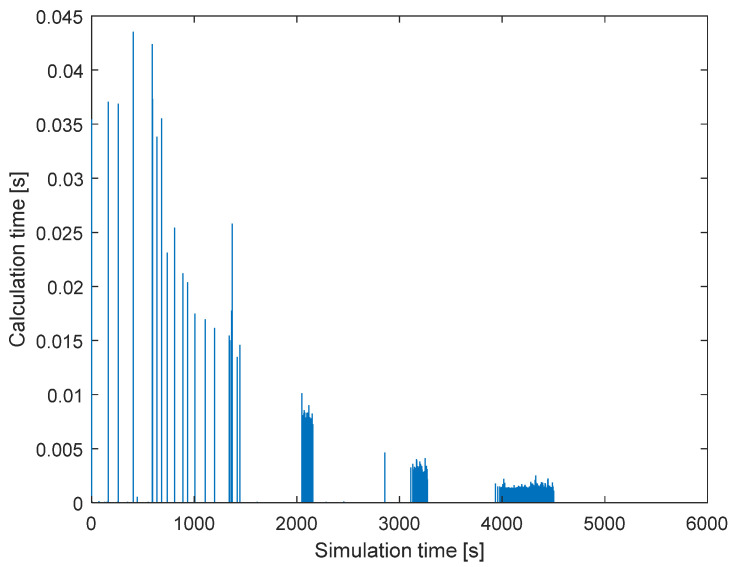
Calculated time-consuming curve in each step.

Experiment 1: 8 UUVs ($U:=\left\{U_{1},U_{2},\cdots ,U_{8}\right\}$) and 40 tasks ($T:=\left\{T_{1},T_{2},\cdots ,T_{40}\right\}$) are set up, and the initial positions of UUVs and tasks are randomly distributed in the simulation area with a communication range constraint of 1 km, i.e., $D_{com\;dis}=1\;\mathrm{km}$. UUV initial position coordinates and task coordinates are shown in Table 5 and Table 6. Simulation experiments are conducted by using the original CBBA (without communication range constraint), the ECBBA (without communication range constraint), the ECBBA (with communication range constraint), and the DECBBA (with communication range constraint) proposed in this paper, respectively. The results are shown in Figure 4 and Figure 5.

In Figure 4, the red dot symbol represents the target position, and the hollow circle symbols and dotted lines of different colors represent the initial positions and movement paths of different UUVs.

Based on the comparison of Figure 4a,b, it can be seen that under the ideal scenario where the communication range is unconstrained, multiple tasks such as $T_{1}$, $T_{2}$, etc. are not assigned to any UUV in the allocation profile obtained from the original CBBA, which makes the global utility and task completion rate lower, and the ECBBA proposed in this paper can complete as many tasks as possible, and the task allocation profile is obviously more reasonable with lower cost and higher global utility.

By comparing Figure 4a–d, we can see that under the scenario with communication range constraint, UUVs can only communicate with other UUVs within their communication range at the initial moment in both the original CBBA and the ECBBA, which leads to some task conflicts that cannot be resolved and multiple UUVs are assigned to the same task, resulting in a serious waste of resources. While in the DECBBA, it is also in the context of communication range constraints, UUVs with task conflicts can communicate when they are close to each other, compare their respective task marginal utility values and resolve the conflicts through communication, and re-plan reasonable paths so as to accomplish more tasks. It can be seen that their planned paths are very close to those derived by the static task allocation algorithm under the ideal scenario of unconstrained communication range, which indicates that the DECBBA is able to overcome the disadvantage of limited communication range.

Figure 5 shows the relationship between the calculation time and simulation time of the DECBBA in Experiment 1. It is easy to see that the time steps with more computation time tend to be more concentrated, as the task bundle reconfiguration phase and conflict resolution phase are only executed when changes occur in the communication network in the DECBBA. Throughout the simulation, the peak computation time does not exceed 0.045 s, and most of the simulation steps are computed in less than 0.01 s, meeting the real-time performance requirements of the algorithm.

**Figure 6:**
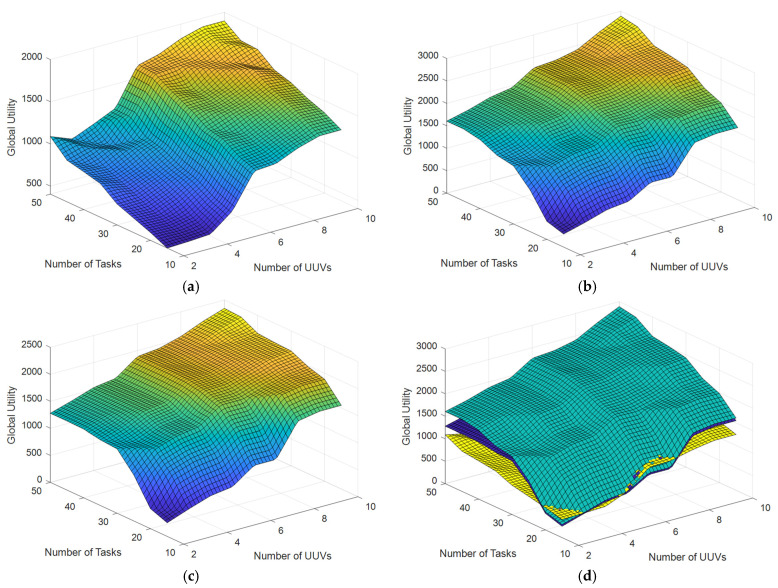
Global utility with different numbers of UUVs and tasks. (**a**) Original CBBA (without communication range constraint). (**b**) ECBBA (without communication range constraint). (**c**) DECBBA (with communication range constraint). (**d**) Comparison of Three Algorithms.

**Figure 7:**
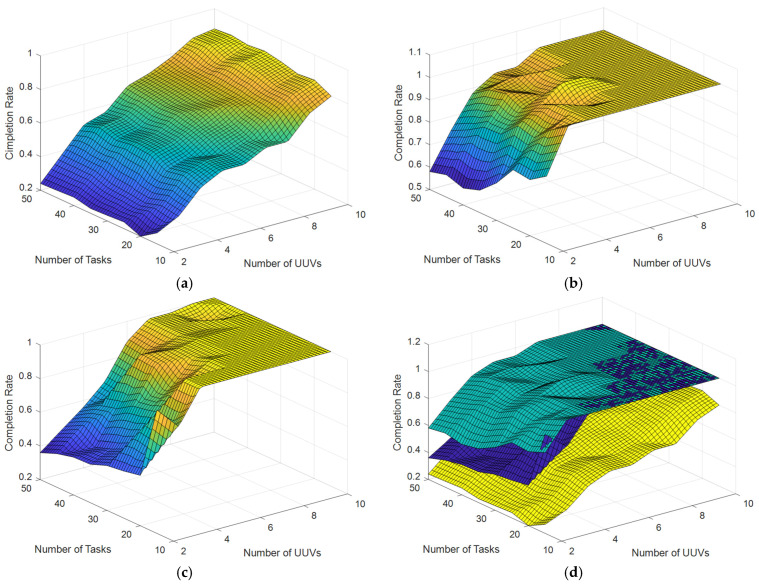
Completion rate with different numbers of UUVs and tasks. (**a**) Original CBBA (without communication range constraint). (**b**) ECBBA (without communication range constraint). (**c**) DECBBA (with communication range constraint). (**d**) Comparison of Three Algorithms.

Experiment 2: Multiple comparison scenarios with $N_{A}=2,3,\cdots ,10$ numbers of UUVs and $N_{T}=10,15,\cdots ,50$ numbers of tasks are set up. The initial positions of UUVs and tasks are randomly distributed in the simulation area with a communication range constraint of 1 km, i.e., $D_{com\;dis}=1\;\mathrm{km}$. The simulation experiments are conducted by using the original CBBA (without communication range constraints), the ECBBA (without communication range constraint), and the DECBBA (with communication range constraint) proposed in this paper, respectively. The results are shown in Figure 6 and Figure 7, Table 7.

Figure 6 shows the global utilities of each of three algorithms under scenarios with different UUVs and tasks number and their comparison. Table 7 lists the global utilities of three algorithms with different UUVs and tasks number. It is easy to see that the global utilities of all three algorithms increase as the number of UUVs and the number of tasks increase. Under the ideal scenario without communication range constraint, the global utility of the original CBBA does not exceed 2000, while the maximum global utility of ECBBA is close to 3000. Additionally, under the scenario with the communication range constraint, the maximum global utility of DECBBA is close to 2500. From the comparison of three algorithms in Figure 6d, it can be seen that global utility of the DECBBA under the scenario with the communication range constraint (blue data) is very close to that of the ECBBA under the ideal scenario without the communication range constraint (green data), and both are significantly higher than that of the original CBBA under the ideal scenario without communication range constraint (yellow data), which proves the superiority of the algorithm proposed in this paper.

Figure 7 shows the task completion success rates of each of the three algorithms under scenarios with different UUVs and tasks number and their comparison. It can be seen that the task completion rates of all three algorithms increase as the number of UUVs increases when the number of tasks is kept constant, while the change in task completion rates is relatively small as the number of tasks increases when the number of UUVs is kept constant. From Figure 7d, it is easy to see that the task completion rate of the original CBBA (yellow data) cannot reach 100% under the ideal scenario without communication range constraint even when the number of UUVs is large enough due to the unreasonable utility function. In contrast, when the number of UUVs is large enough, the task completion rate of both ECBBA (green data) and DECBBA (blue data) can reach 100%, i.e., all tasks can be completed, which proves the effectiveness of the algorithm proposed in this paper.

## 6. Conclusions

Aiming at the task allocation problem of heterogeneous UUV swarms, this paper proposes a dynamic task allocation algorithm that extends CBBA. The algorithm considers the multiple assignment problem that each UUV can individually complete multiple tasks, constructs a “UUV-task” matching matrix and designs new marginal utility, reward and cost functions for the influence of time, path and UUV voyage. Furthermore, in view of the unfavorable factors that restrict the underwater acoustic communication range between UUVs in the real environment, our algorithm completes UUV swarms dynamic task allocation with optimization in load balance indicator by the update of the UUV individual and the task completion status in the discrete time stage. In this paper, the superiority and effectiveness of the proposed algorithms is demonstrated by simulation experiments. The simulation results show that the proposed ECBBA in this paper has more reasonable task allocation profiles, higher global utility and higher task completion rate compared with the original CBBA. The performance indicators (including global utility and task completion rate) of the proposed DECBBA under the scenario with the communication range constraint can be very close to the ECBBA under the ideal scenario without the communication constraint scenario. In short, the algorithm proposed in this paper achieves dynamic conflict-free task allocation for multi-UUV swarms with excellent performance. However, the research in this paper is still deficient in the following aspects, which can be extended and improved: (1) when the number of UUVs or the number of tasks to be completed is large, the communication traffic between UUVs is large based on the consistency requirement of the algorithm, which requires high computing resources and communication bandwidth. We plan to reduce the communication traffic between UUVs as the focus of future research. (2) When the UUV swarm completes the task in a large area, the communication delay will limit the task conflict resolution phase between UUVs due to the limitation of hydro-acoustic communication speed. We plan to add communication delay as a constraint to optimize the algorithm in future simulation and underwater real environment experiments. (3) The speed and success rate of reaching consensus between UUVs will also be affected when there is an unexpected condition of failure by individual UUVs, so the single point of failure problem is also one of the factors worth considering in future research.

## Figures and Tables

**Table 1:** Conflict resolution rules.

$z_{kj}$	$z_{ij}$	$U_{i}\;\mathrm{Action}$
k	i	$\mathrm{if}\;y_{kj}>y_{ij}$, update
	k	update
	m∉{k,i}	$\mathrm{if}\;y_{kj}>y_{ij}$$\;\mathrm{or}\;s_{km}>s_{im}$, update
	∅	update
i	i	leave
	k	reset
	m∉{k,i}	$\mathrm{if}\;s_{km}>s_{im}$, reset
	∅	leave
m∉{k,i}	i	$\mathrm{if}\;y_{kj}>y_{ij}$$\;\mathrm{or}\;s_{km}>s_{im}\;$, update
	k	$\mathrm{if}\;s_{km}>s_{im}$, update, otherwise, reset
	m	$\mathrm{if}\;s_{km}>s_{im}$, update
	n∉{k,i,m}	$\mathrm{if}\;s_{km}>s_{im}$$\;\mathrm{and}\;s_{kn}>s_{in}$, update
		$\mathrm{if}\;y_{kj}>y_{ij}$$\;\mathrm{and}\;s_{km}>s_{im}$, update
		$\mathrm{if}\;s_{km}<s_{im}$$\;\mathrm{and}\;s_{kn}>s_{in}$, reset
	∅	$\mathrm{if}\;s_{km}>s_{im}$, leave
∅	i	leave
	k	update
	m	$\mathrm{if}\;s_{km}>s_{im}$, update
	∅	leave

**Table 2:** UUV parameters.

UUV Type	$\mathrm{Velocity}\;V_{i}\;(m/s)$	$\mathrm{Voyage}\;D_{i}\;(m)$	$\mathrm{Fuel}\;\mathrm{Consumption}\;\mathrm{Per}\;\mathrm{Unit}\;\mathrm{Mile}\;F_{i}\;(/m)$
portable	2	3000	1
light	3	5000	2
heavy	5	10,000	3

**Table 3:** Task parameters.

Task Type	$\mathrm{Duration}\;t_{j}\;(s)$	$\mathrm{Static}\;\mathrm{Reward}\;{\bar{r}}_{j}$	$\mathrm{Time}-\mathrm{Discounting}\;\mathrm{Factor}\;\lambda_{j}$
detect	300	2000	0.005
track	600	5000	0.01
rescue	1200	10,000	0.02

**Table 4:** UUV and task matching types.

		Task Type		
		Detect	Track	Rescue
UUV Type	portable	Y	N	N
	light	Y	Y	N
	heavy	N	Y	Y

**Table 5:** UUV initial position.

UUV Id	Initial Position Coordinates (m)
$U_{1}$	(8734, 9685)
$U_{2}$	(8692, 5309)
$U_{3}$	(2327, 114)
$U_{4}$	(4305, 4024)
$U_{5}$	(5227, 4784)
$U_{6}$	(5554, 5434)
$U_{7}$	(7615, 7124)
$U_{8}$	(6197, 4261)

**Table 6:** Task position.

Task Id	Position Coordinates (m)	Task Id	Position Coordinates (m)	Task Id	Position Coordinates (m)	Task Id	Position Coordinates (m)
$T_{1}$	(2891, 9739)	$T_{11}$	(4325, 2702)	$T_{21}$	(7312, 9397)	$T_{31}$	(7065, 5106)
$T_{2}$	(3338, 2188)	$T_{12}$	(8011, 6382)	$T_{22}$	(9433, 3747)	$T_{32}$	(8301, 7207)
$T_{3}$	(658, 9828)	$T_{13}$	(687, 6036)	$T_{23}$	(5958, 6620)	$T_{33}$	(8663, 2034)
$T_{4}$	(1279, 3221)	$T_{14}$	(7956, 320)	$T_{24}$	(5106, 4709)	$T_{34}$	(2985, 4758)
$T_{5}$	(709, 2248)	$T_{15}$	(4555, 7902)	$T_{25}$	(89, 679)	$T_{35}$	(4936, 6077)
$T_{6}$	(3936, 8962)	$T_{16}$	(9886, 5840)	$T_{26}$	(4348, 4315)	$T_{36}$	(8189, 5277)
$T_{7}$	(3455, 9848)	$T_{17}$	(389, 4464)	$T_{27}$	(1859, 5297)	$T_{37}$	(9392, 7234)
$T_{8}$	(287, 3517)	$T_{18}$	(1882, 6271)	$T_{28}$	(5991, 7209)	$T_{38}$	(1788, 6197)
$T_{9}$	(3810, 7642)	$T_{19}$	(2153, 1510)	$T_{29}$	(3064, 4044)	$T_{39}$	(5623, 7634)
$T_{10}$	(9388, 3197)	$T_{20}$	(5361, 1004)	$T_{30}$	(8228, 5871)	$T_{40}$	(8067, 2986)

**Table 7:** Global utility with different numbers of UUVs and tasks.

	CBBA			ECBBA			DECBBA		
	$N_{T}=20$	$N_{T}=30$	$N_{T}=50$	$N_{T}=20$	$N_{T}=30$	$N_{T}=50$	$N_{T}=20$	$N_{T}=30$	$N_{T}=50$
$N_{U}=3$	488.9046	663.0193	987.1419	944.6565	1321.439	1721.228	886.2122	1225.167	1400.739
$N_{U}=6$	1110.785	1277.934	1647.512	1447.621	1967.616	2263.104	1365.22	1819.503	1888.382
$N_{U}=10$	1356.783	1530.076	1897.551	1984.603	2430.998	2889.624	1888.186	2125.762	2344.501

## Data Availability

Not applicable.

## Nomenclature

$N_{U}$	number of UUVs
$N_{T}$	number of tasks
$U_{i}$	the ith UUV
$T_{j}$	the jth task
${\mathrm{Match}}_{ij}$	the UUV and task matching matrix
f	global objective function
$A_{i}$	binary decision vector of $U_{i}$
$P_{i}$	task completion path sequence of $U_{i}$
$B_{i}$	maximum number of tasks that $U_{i}$ can complete
$r_{ij}\left(A_{i},P_{i}\right)$	$T_{j}$’s utility obtained when it is performed by $U_{i}$ in path $P_{i}$
D(·)	path cost
$D_{i}$	maximum voyage of $U_{i}$
$b_{i}$	task sequence of $U_{i}$
$p_{i}$	path of $U_{i}$
$S_{i}^{p_{i}}$	utility obtained by $U_{i}$ by performing $T_{j}$ along $p_{i}$
$p_{i}{⊕}_{n}\left\{j\right\}$	task $T_{j}$ inserted into the nth position in $p_{i}$
$S_{i}^{p_{i}{⊕}_{n}\left\{j\right\}}$	new utility after inserting $T_{j}$ into the nth position in $p_{i}$
$\Delta S_{ij}$	marginal utility value
$R_{j}^{p_{i}}$	marginal reward function
${\bar{r}}_{j}$	static reward value for task $T_{j}$
$\lambda_{j}$	time-discounting factor for task $T_{j}$
$\tau_{i}^{j}\left(p_{i}\right)$	time $U_{i}$ takes to travel along path $p_{i}$ to task $T_{j}$
$T_{m}$	task added to the task sequence before $T_{j}$ but located after $T_{j}$ in the path
$\Delta R_{m}^{p_{i}}$	marginal increment of $T_{m}$
$cost_{T_{j}}^{p_{i}}$	marginal cost function
$F_{i}$	fuel consumption per unit mile of $U_{i}$
s	timestamp vector
G	communication network
